# SinglePass electrocautery device for closure of lung and mediastinal biopsy: a case series of 5 patients

**DOI:** 10.3389/fonc.2026.1874234

**Published:** 2026-06-30

**Authors:** Tust Techasith, Abhishek Jairam, Benjamin Park, Avinash Mesipam, Christopher Baker, Brian Cristiano, Thomas Velling, Trushar Patel, Behroz Oftadeh, Quan Dang, Lindsey Young, Vincent Nguyen, Kristen Raust, Matthew Roughley, Nathan Nguyen, Yurtizi Chavez, Alexander S. Misono

**Affiliations:** Hoag Memorial Hospital Presbyterian, Newport Radiology, Newport Beach, CA, United States

**Keywords:** bleeding, case report, core needle biopsy, lung nodules biopsy, mediastinal mass biopsy, percutaneous lung nodules biopsy, pneumothorax

## Abstract

**Introduction:**

Percutaneous computed tomography (CT)-guided biopsy, including coaxial core needle biopsy (CCNB), has become the primary and preferred method for obtaining tissue samples in diagnosing or characterizing thoracic lesions, often in the lung or mediastinum. Despite advances in imaging and various approaches in reducing bleeding in the biopsy tract, pneumothorax and perilesional or lesional hemorrhage remain at the forefront of potentially serious complications. The increased risk that comes with requirements for additional tissue samples, both in size and in number, in the management of lung cancer is also a concern. Subsequent interventions may be required if significant pneumothorax or high-grade hemorrhage occurs and while death is a possible outcome with these procedures, it is rare.

**Case presentation:**

This report of a series of five image-guided CCNB cases focused on lung and mediastinal mass lesions in which hemostasis and biopsy tract closure were obtained with the SinglePass Kronos electrocautery device. All biopsy procedures were successful, and no complications were reported through 30 days post-procedure. Imaging confirmed the absence of post-procedure pneumothorax and bleeding complications in all five cases.

**Conclusion:**

The SinglePass electrocautery device is a potentially valuable tool in minimizing pneumothorax and hemorrhagic complications in percutaneous lung and mediastinal mass CCNB procedures.

## Introduction

Percutaneous, computed tomography (CT) guided core needle biopsy plays a crucial role and is the preferred method in the diagnostic work−up of thoracic and pulmonary lesions, offering high diagnostic yield with minimally invasive access. It is also more cost-effective than surgical or bronchoscopic methods (1). For lung biopsies, current literature reports a diagnostic sensitivity of 85−95%, with malignancy−detection rates between 92% and 97%, complication rates for pneumothorax range from 12 to 45%, and pulmonary hemorrhage rates from 4 to 27% (2). Similarly, mediastinal procedures are also highly successful as a recent meta-analysis reported pooled diagnostic yield and accuracy of 92% and 94% (3). Despite advances in imaging guidance, needle system design, and tract sealing and “plugging” techniques (e.g., gels, slurries, and coils), pneumothorax and perilesional hemorrhage remain serious complications (2, 4–6). Air embolism is also a risk, but rare (<0.1%) (7). Death also occurs rarely but is usually related to pre-existing comorbidities rather than the biopsy procedure (8).

With the increase in the incidence of chest tumors and the widespread use of CT detecting more pulmonary nodules and intrathoracic masses, coaxial core needle biopsy (CCNB) plays an increasingly important role in evaluating these lesions (4). Advances in the management of malignancies such as lung cancer require greater amounts of tissue for tumor genomic profiling and characterization creating a need to obtain more samples per procedure (9). Increased sampling would logically result in an increase in complications, but the current literature is mixed on multiple sample complication rates (2, 9). Obtaining more tissue through larger gauge biopsy needles (e.g.,16-gauge) has also been an effective solution to satisfy increased sample demand, but comes with a clear caution for increased pneumothorax risk (10).

Complications are also influenced by factors such as needle path, lung parenchymal characteristics, underlying coagulopathy, and lesion size, depth, and location (11–15). These require effective management and vigilance during every phase of the biopsy procedure such as putting preventative strategies in place prior to the biopsy, making real-time intraprocedural adjustments and early recognition of events during the post-procedural period (2). Mediastinal total and major complication rates are typically lower, 13% and 2% respectively, particularly when a transpleural approach is avoided (3).

A case series of lung and thoracic lesion biopsies performed with the SinglePass Kronos electrocautery device is presented to evaluate its safety, feasibility and early outcomes compared to lung biopsy complication rates published in the literature. The SinglePass device has shown promise in managing needle tract hemostasis in two small series of liver and kidney biopsy cases (16, 17).

## Materials and methods

Percutaneous CCNB was performed using CT guidance in an outpatient setting for various lung and mediastinal mass tissue assessments in five patients. At this institution, lung lesion biopsies are typically performed using Bard Max-Core Disposable Core Biopsy Instruments (Becton Dickinson, Inc., Franklin Lakes, NJ) in either 19/20-gauge or 17/18-gauge sizes. Historically, most biopsies have been performed with 19/20-gauge systems due to institutional practice patterns. For these cases, 17/18-gauge devices were employed. Following the biopsy, the SinglePass electrocautery device (SinglePass, Inc., Lake Forest, CA) was applied along the needle tract to achieve both hemostasis and air leak or pneumothorax control. The SinglePass device (see **Figure 1**) is a disposable, battery-powered electrocautery instrument designed for bleeding control after core needle biopsies of organ tissue, including the liver, kidney, and lung. It features an ergonomic handle that activates and heats the tip of a probe, which reaches temperatures up to 85˚C to cauterize tissue.

**Figure 1:**
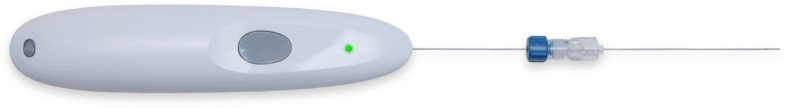
SinglePass electrocautery device.

Prior to performing the biopsy, the SinglePass device excursion depth was set by aligning the biopsy guide needle with the SinglePass device to ensure complete cauterization of the biopsy tract. Once the SinglePass device was inserted and connected to the depth gauge, the assembly was advanced until the SinglePass device tip was exposed to match the intended biopsy gun length, usually between 10 and 22 mm. The depth gauge was then tightened securely onto the SinglePass probe, after which the guide needle was removed and the CCNB procedure performed.

After the biopsy, the guide needle was left in place, and the SinglePass device was inserted matching the depth of the tissue extractor. With the guide needle fastened to the SinglePass device, the power button was activated to heat the probe tip and after approximately 10 seconds, the combined assembly was slowly withdrawn cauterizing the needle channel thus establishing hemostasis and air-leak or pneumothorax control. The needle was pulled at a rate of approximately 5 mm per second to seal the tract and visceral pleural surface while minimizing any cauterization impact on surrounding intercostal structures such as nerves.

Any observed procedure-related hematoma was monitored for stability via serial CT imaging immediately post-biopsy over a 5- to 10-minute period to document bleeding cessation. Following the procedure, patients received calls from interventional radiology nursing 24 to 48 hours post-procedure with successful interactions documented in the patient’s electronic medical record (EMR) and their EMR was also retrospectively reviewed for study-related events through 30 days post-CCNB. Patients in this series were treated within a healthcare network with a largely closed, internally referred patient population and as such, patients with procedure-related events tend to routinely return to an in-network urgent care or emergency department.

Informed consent to perform the procedure was obtained from each patient.

## Results

A summary of the cases that comprise this series is provided in **Table 1** below:

**Table 1:** Case summaries

Case #	Age/sex	History	Imaging	Medication	Diagnosis	Treatment
1	94/F	Lung nodules	Upper right lobe mass on CT	Eliquis	Extensive necrosis	Continued surveillance
2	85/F	Lung nodules	Upper right lobe mass on CT	None	Low-grade B-cell lymphoma	Rituximab infusions & radiation
3	75/M	Persistent lung nodule	Lower right lobe nodule on CT	None	Focal congestion, scarring, and mild chronic interstitial inflammation	Continued surveillance
4	79/M	Lung cancer, systemic & radiation treatment	Persistent left upper lobe airspace opacity	None	Fibrosis and elastosis, presumed scarring due to treatment	Continued surveillance
5	40/F	No relevant history	10cm mediastinal mass involving/abutting left upper lobe on MRI	None	T-cell infiltrate consistent with thymoma	Operative resection, chemotherapy, and radiation

### Case #1

A 94-year-old female patient with a history of lung nodules underwent CCNB following identification of a lung mass in the right upper lobe of the lung on CT imaging (**Figure 2a**). The patient was taking Eliquis, which was halted prior to the procedure per institutional guidelines and her pre-procedure labs were unremarkable.

**Figure 2:**
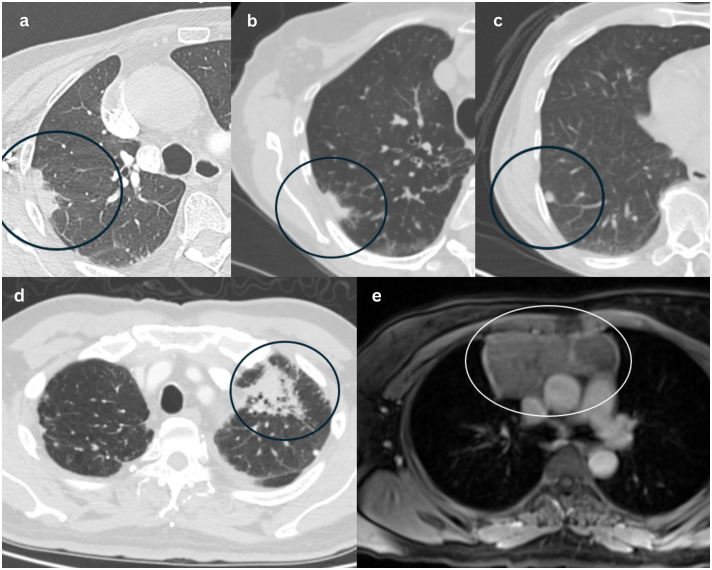
Pre-procedure images showing target lesions for each case. **(a)** Case #1 (black circle); **(b)** Case #2 (black circle); **(c)** Case #3 (black circle); **(d)** Case #4 (black circle); **(e)** Case #5 (white circle).

Core samples were obtained under CT guidance in a right lateral decubitus position with a posterior approach. The biopsy procedure, followed by SinglePass electrocautery, was completed without complications and the patient was discharged 1 hour post-procedure. Immediately post-procedure, CT imaging confirmed the expected minor peri-nodular parenchymal changes, and the absence of significant bleeding complications including a clearly defined cautery track (**Figures 3a, b**). This imaging hinted at a possible minimal pneumothorax that was definitively excluded upon subsequent clinical and imaging evaluation, including a chest X-ray confirming no pneumothorax at discharge (**Figure 4a**).

**Figure 3:**
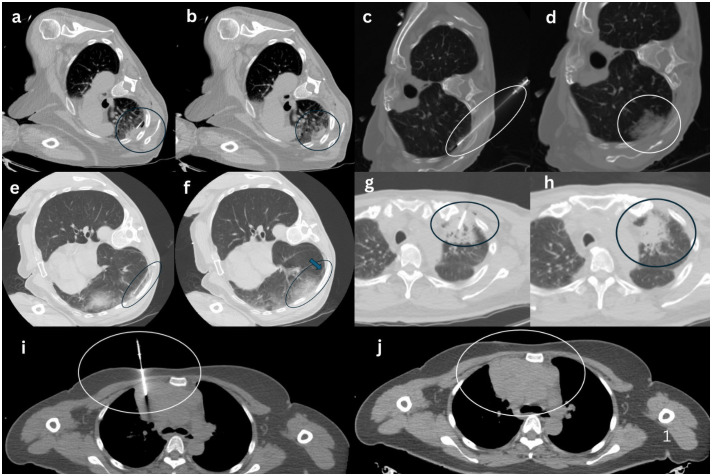
Peri- and post-procedure CT images. Case #1 –**(a)** radiopaque core needle (black circle); **(b)** post-procedure biopsy site (black circle) with expected peri-nodular hemorrhage and no pneumothorax; Case #2 – **(c)** core needle *in situ* (white circle); **(d)** post-procedure biopsy site with expected peri-nodular hemorrhage and no complications; Case #3 – **(e)** access needle (black circle); **(f)** post-procedure biopsy site (black circle) with expected peri-nodular hemorrhage and linear opacity of cautery tract (blue arrow); Case #4 – **(g)** core needle *in situ* (black circle); **(h)** post-procedure biopsy site (black circle) showing no complications; Case #5 – **(i)** core needle access (white circle); **(j)** post-procedure biopsy site (white circle) showing no complications.

**Figure 4:**
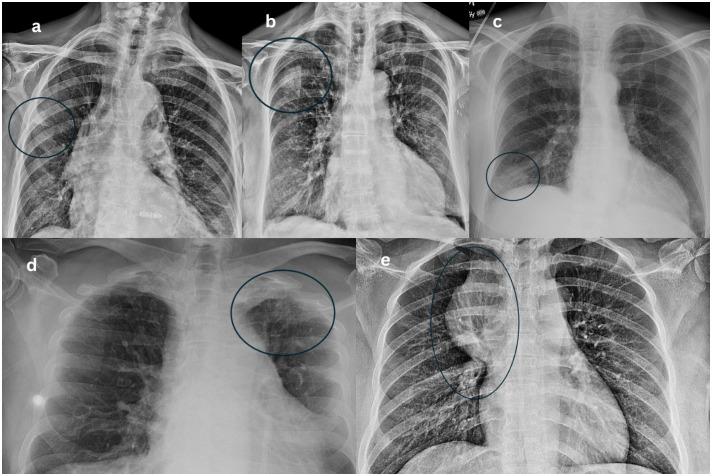
Post-procedure chest x-rays demonstrating the absence of pneumothorax (biopsy site identified by a black circle) for each case: **(a)** Case #1; **(b)** Case #2; **(c)** Case #3; **(d)** Case #4; **(e)** Case #5.

There were no complications reported in the 30 days following the procedure.

The pathology results showed lung parenchyma with extensive necrosis suspected to be secondary to infarct. Special stains did not reveal any microorganisms, and no evidence of malignancy was identified. She has continued to follow up with pulmonary medicine. Repeat imaging at 3 months demonstrated no significant change in the lung mass. Continued imaging surveillance is planned.

### Case #2

An 85-year-old female patient with a history of a lung nodule underwent CCNB following identification of a lung mass in the right upper lobe of the lung on CT imaging (**Figure 2b**). The patient was not taking antiplatelet or anticoagulant medications, and all pre-procedure laboratory testing was within normal limits.

Core samples were obtained using a posterior approach in a right lateral decubitus position under CT guidance. The biopsy procedure (**Figure 3c**), followed by SinglePass electrocautery was completed without complications and the patient was discharged 1-hour post-procedure. Immediately post-procedure CT imaging confirmed typical post-biopsy peri-nodular parenchymal changes, no pneumothorax, and the absence of significant bleeding complications (**Figure 3d**). Chest X-ray confirmed no pneumothorax at discharge (**Figure 4b**). There were no complications reported in the 30 days following the procedure.

Pathology results concluded that this mass was consistent with low-grade B-cell lymphoma. After evaluation by oncology and radiation oncology, she entered therapy with rituximab infusions in conjunction with external beam radiation therapy.

### Case #3

A 75-year-old male patient with a history of a persistent lung nodule on multiple scans underwent CCNB following identification of a 1.2 cm lung nodule in the right lower lobe of the lung on CT imaging (**Figure 2c**). The patient was not taking antiplatelet or anticoagulant medications, and all pre-procedure laboratory testing was within normal limits.

Core samples were obtained in a right lateral decubitus position using a posterior approach under CT guidance. The biopsy procedure (**Figure 3e**), followed by SinglePass device electrocautery, was completed without complications and the patient was discharged 1-hour post-procedure. Immediately post-procedure CT imaging confirmed the expected post-biopsy peri-nodular changes, no pneumothorax, and the absence of bleeding complications (**Figure 3f**). Chest X-ray confirmed no pneumothorax at discharge (**Figure 4c**).

There were no complications reported in the 30 days following the procedure.

Pathology demonstrated lung tissue with focal congestion, scarring, and mild chronic interstitial inflammation. No evidence of neoplasm was identified. Follow up imaging under the care of pulmonary medicine demonstrated the nodule had decreased in size at 3 months. The patient continues to undergo surveillance imaging.

### Case #4

A 79-year-old male patient with a history of known lung cancer and a persistent left upper lobe airspace opacity despite systemic therapy and radiation underwent CCNB (**Figure 2**). The patient was not taking antiplatelet or anticoagulant medications, and all pre-procedure laboratory testing was within normal limits.

Core samples were obtained under CT guidance using an anterior approach in a supine position. The biopsy procedure (**Figure 3g**), followed by SinglePass electrocautery, was completed without complications and the patient was discharged 1-hour post-procedure. Immediately post-procedure, CT imaging confirmed the no pneumothorax and no bleeding complications (**Figure 3h**). Chest X-ray confirmed no pneumothorax at discharge (**Figure 4d**).

There were no complications reported in the 30 days following the procedure.

Pathology demonstrated fibrosis and elastosis, suggestive of a pulmonary scar tissue with no evidence of malignancy. Contemporaneous PET CT revealed low FDG avidity along the radiation margins, felt to be secondary to treatment changes. The patient continues to undergo surveillance imaging under the care of oncology.

### Case #5

A 40-year-old female patient underwent CCNB following identification of a 10cm mediastinal mass involving or abutting the left upper lobe of the lung on MRI imaging (**Figure 3e**). The patient was not taking antiplatelet or anticoagulant medications, and all pre-procedure laboratory testing was within normal limits.

Core samples were obtained in a supine position using an anterior approach under CT guidance. The biopsy procedure (**Figure 3i**), followed by SinglePass device electrocautery, was completed without complications and the patient was discharged 30 minutes post-procedure. Immediately post-procedure, CT imaging confirmed no complications including no evidence of bleeding or pneumothorax (**Figure 3j**). Chest X-ray confirmed no pneumothorax at discharge (**Figure 4e**).

There were no complications reported in the 30 days following the procedure.

Pathology results were predominantly T-cell infiltrate which is most consistent with thymoma. The patient has subsequently undergone operative resection, chemotherapy, and radiation therapy.

## Discussion

Pneumothorax and bleeding complications remain an ongoing concern in intrathoracic percutaneous needle biopsies despite innovations to manage the biopsy tract. Pneumothorax presents the most common risk faced during intrathoracic biopsy, with more severe cases requiring chest tube insertion. A recent study looking at data from over 2,500 CT-guided lung biopsy patients over a 30-year period showed a chest tube insertion rate of 6.7% with age, patient position, and severe emphysema being the most predictive factors (18). Electrocautery of the visceral pleural surface may decrease the risk of pneumothorax formation by sealing the puncture hole.

Hemorrhage is the second most frequent complication when performing CCNB in lung tissue requiring effective management and preventive strategies. While hemorrhage can be a complication, it is also so frequently seen that most interventionalists expect some degree of pulmonary hemorrhage during procedures However, in more severe cases, hemoptysis can occur, affecting up to 3-4% of patients (2, 19). Smaller lesions (<2 cm), longer needle courses (>4 cm), no pleural effusion, and multiple punctures were reported as having the highest impact on bleeding rates (2). Bleeding risk increased when sampling smaller lesions due to the fine adjustments often required to access the lesion. Most significant, relative to SinglePass device use, is that minor alveolar hemorrhage along the needle tract accounts for 86% of pulmonary bleeding (2). Sealing the biopsy tract quickly may minimize these serious complications.

Despite proximity to central organs and vasculature, complication rates of mediastinal CCNB are generally lower. One large, single-center series reported pneumothorax rates as low as 1.5% and bleeding complications below 1% with only one report of a symptomatic, moderate-sized pneumothorax and chest tube insertion (20). These relatively low rates may still hold clinical significance, and electrocautery may provide a viable preventative solution.

Using thermal energy to manage bleeding in surgical procedures has been in practice for over 100 years (21). As related to the lungs, microwave ablation to treat lung tumors has been successful in reducing the size and speed of pneumothorax formation and reducing pleural effusions showing air sealing effects of thermal energy (22). Similarly, although significantly different from a needle biopsy, electrocautery has been shown to be safe and acceptable for lung sublobular resection (23). Therefore, the SinglePass electrocautery device makes for a logical approach to seal off the affected biopsy tract and reduce potential complications related to CCNB.

Many patients undergoing CCNB, including those with lung and thoracic lesions, are taking blood thinning medications such as anticoagulation. Anticoagulants can increase the risk of pulmonary hemorrhage (24). It is notable that the patient reported in Case #1 was on apixaban prior to their biopsy and had no bleeding complications. Further investigation of the benefits of electrocautery in lung biopsy in patients with disordered coagulation or exposure to antiplatelet or anticoagulation medication is warranted.

The SinglePass device is a novel electrocautery device that was well-tolerated and has demonstrated rapid and durable needle tract hemostasis during this small, early case series of lung and mediastinal CCNB procedures. While more data and comparative evaluation is needed, the absence of complications in these 5 cases demonstrates the potential feasibility of this new technology.

This early case series suggests the SinglePass device - a disposable electrocautery device - can be used post-lung and mediastinal mass biopsy safely, with promising technical and clinical results. There was no evidence of pneumothorax or bleeding complications including hemoptysis in any of the 5 cases despite concomitant anticoagulant use in one patient. With a plethora of factors affecting the outcomes of lung and mediastinal mass biopsy, larger controlled studies with longer follow-up are needed to determine if these outcomes are reproducible. Managing and preventing further complications by sealing the biopsy tract with the SinglePass device demonstrates potential.

## Data Availability

The raw data supporting the conclusions of this article will be made available by the authors, without undue reservation.
